# Maternal Stress Mediates Association of Infant Socioemotional Development with Perinatal Mental Health in Socioeconomically Vulnerable Peruvian Settings

**DOI:** 10.3390/ijerph21070844

**Published:** 2024-06-28

**Authors:** Magaly Nóblega, Olenka Retiz, Juan Nuñez del Prado, Ramón Bartra

**Affiliations:** Department of Psychology, Pontifical Catholic University of Peru, Lima 15088, Peru; olenka.retiz@pucp.pe (O.R.); j.nunezdelprado@pucp.pe (J.N.d.P.); ramon.bartra@pucp.pe (R.B.)

**Keywords:** perinatal, maternal mental health, parental stress, socioemotional development

## Abstract

Increased maternal mental health during the perinatal period has been widely associated with a variety of positive outcomes for both mothers and infants. However, no studies in Peru have yet focused on studying maternal mental health and related psychological variables during this stage. Thus, the aim of this study was to test a model to associate a mother’s parental stress with infant socioemotional difficulties and maternal mental health. The sample included 988 mothers of infants aged 6 to 18 months from Peru, all from socioeconomically vulnerable settings. The findings showed that infant socioemotional difficulties were associated with poorer maternal mental health through the mother’s parental stress (*χ*^2^(7) = 28.89, *p* < 0.001, CFI = 0.98, RMSEA = 0.06, SRMR = 0.03). These results provide a better understanding of the key elements associated with maternal mental health during the perinatal period in Peru and offer valuable insights for developing interventions and support strategies for socioeconomically vulnerable mothers and their young children.

## 1. Introduction

The perinatal period, spanning from pregnancy to the first two years postpartum [1], is considered a milestone in a woman’s life with significant long-term implications for her mental health [2] and her offspring’s development [3]. At the same time, it is a stage of psychological risk; approximately one in five women will experience some perinatal mental health difficulty [4]; this rate can be twice as high in low- and middle-income countries [5] such as Peru. Despite the widely acknowledged importance of perinatal maternal mental health [6], there is a lack of Latin American and Peruvian studies aimed at identifying its associated factors in both the general populations and those living in socioeconomic vulnerability. Studying these variables would be highly beneficial for identifying intervention targets and establishing public policy guidelines that promote the well-being of women and their families, particularly in less privileged sectors where maternal mental health difficulties are often more pronounced [7].

### 1.1. Maternal Perinatal Mental Health

The perinatal period entails a transition time during which women must adapt to biological, psychological, and social changes associated with the birth of their child and its subsequent care [8]. These changes predispose women to a higher risk of experiencing psychopathological symptoms and new or recurrent episodes of mental disorders [3,9,10].

Mental health problems during the perinatal period have been linked to negative outcomes for mothers and their children [11]. For mothers, these problems can increase the risk of persistent psychopathological symptoms over time, as well as a higher risk of suicide, substance abuse, and interpersonal violence [12,13,14,15,16]. For children, maternal mental health problems have been associated with physical health difficulties during the first year of life [17], as well as socioemotional and psychological problems [18]. These adverse outcomes for the child can be attributed to a reduced maternal capacity for mentalization and sensitivity, which deteriorates mother–infant interactions and impacts children’s development [19,20,21].

Rates of symptomatology during the perinatal period are generally higher among women of low socioeconomic status and among racial/ethnic minorities [5,22]. These women may face significant obstacles in accessing basic resources such as specialized medical care and treatment for mental disorders, increasing the risk of depression and other mental health problems [23,24,25,26]. Furthermore, the lack of social support and pressure to meet daily demands in a context of scarce resources can contribute to greater isolation and emotional vulnerability in these populations [6,22].

### 1.2. Children, Childcare and Maternal Perinatal Mental Health

It has been found that child characteristics, such as the presence of difficulties in their socioemotional development, are associated with maternal mental health. In young children, these difficulties can manifest as habitual and high irritability, inflexibility to changes, and difficulty in following routines [27]. In some cases, the irritability can be caused by infants’ incipient emotion regulation [28,29]. Children may have difficulties following different routines, specifically sleep routines, which manifest as a “difficult temperament” that limits their ability to self-soothe during the night [30,31]. Another manifestation of difficulties in socioemotional development is inflexibility to novelty, evidenced by excessive fear of unfamiliar people and situations [27], even when the unfamiliar person displays positive affection and synchronizes their actions with the infant [32]. These difficulties tend to be greater in socioeconomically vulnerable contexts, which may be partly due to the reduced quality of parental caregiving [33].

Although previous research has suggested a bidirectional relationship between infant socioemotional development problems and maternal mental health [34], most studies have emphasized the effect of perinatal mental health difficulties on the development of infants’ socioemotional problems [35,36,37], and fewer studies have addressed the effect of children’s socioemotional difficulties on maternal mental health [38,39,40,41,42]. These few studies have found that the presence of problems in children’s socioemotional development can lead mothers to experience feelings of inadequacy, guilt, worry, and frustration, which in turn increase levels of mental health problems [40,41]. Ultimately, a child’s difficulties may affect the mother–child relationship, leading to feelings of emotional disconnection and alienation in the mother, which also contributes to the further deterioration of her mental health [38,39,42].

Additionally, children’s difficulties could contribute to mothers’ parental stress [43] and the aversive psychophysiological responses that arise from attempts to adapt to the demands of motherhood [44]. The perception of parenting as more overwhelming and stressful tends to be higher during the perinatal period compared to other times of motherhood [45], and there is evidence that it is more salient in vulnerable groups of mothers, for example, those with lower educational levels due to less access to expert support and other reliable sources to respond to difficult parenting situations [46]. In addition, it has been found that high levels of maternal parenting stress are associated with a greater risk of mental health difficulties [47,48,49,50] and exacerbate maternal psychopathological symptoms [51]. Based on these considerations, it is possible to hypothesize a mediation of parental stress in the relation between socioemotional development difficulties and maternal perinatal mental health.

### 1.3. Maternal Perinatal Mental Health in Peruvian Socioeconomically Vulnerable Settings

In recent years, empirical studies on perinatal maternal mental health have been conducted in North America, Europe, Asia, and Africa [2,5,13]. However, research in Latin America remains scarce [52,53].

Specifically, in Peru, existing epidemiological studies show that mothers represent around 64.4% of the female population, with an average age at the birth of their first child of 22.4 years. Approximately 77% of them have completed only primary or secondary education [54]. Previous research has shown that most Peruvian mothers live in poverty due to limited access to educational development opportunities [55]. This situation is often normalized by sociocultural standards [56] that assume women’s main tasks are household work and childcare while men are providers of economic resources [57].

Difficulties in accessing adequate education are associated with other multidimensional poverty problems faced by the mothers, such as informal and precarious jobs with low pay and unstable work environments. This situation further complicates the mothers’ ability to overcome poverty, establishing a pernicious cycle that is difficult to break [58]. Additionally, Peruvian mothers from socioeconomically vulnerable settings face limitations in access to nutritious food, contributing to health problems [59]. This is coupled with a lack of access to adequate healthcare services due to economic restrictions or geographical distance [60]. Lastly, these mothers often reside in overcrowded housing with limited access to basic services such as clean water, sanitation, and electricity, increasing their vulnerability to various diseases and hazards [61]. Regarding public safety, crime, and drug consumption in public areas can create an atmosphere of insecurity that further complicates their situation [62].

Other studies conducted in Peru have reported that disorders such as depression during the perinatal period are associated with living in a socioeconomically vulnerable or violent context [63,64,65]. However, to our knowledge, no study has specifically addressed the relationship between perinatal maternal mental health and psychological variables associated with parenting (e.g., parental stress) and child characteristics (e.g., socioemotional development difficulties).

## 2. Materials and Methods

**Figure 1:**
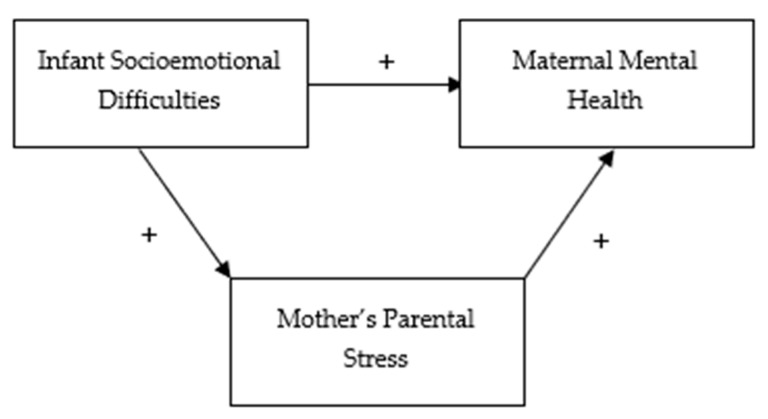
Theoretical proposed model. Note. A plus sign (+) indicates a hypothesized positive association.

The present study aimed to analyze the associations between maternal parental stress and infant socioemotional development difficulties as variables related to maternal perinatal mental health in a socioeconomically vulnerable Peruvian sample. Our goal was to verify whether parental stress mediates the association between infant socioemotional development difficulties and maternal perinatal mental health, thereby confirming the model depicted in Figure 1.

### 2.1. Participants and Procedure

The study population was mothers of infants receiving an intervention to improve infant development in socioeconomically vulnerable children. These mothers came from four regions of Peru.

Since the participants were mothers who voluntarily agreed to participate, this study used convenience sampling. Thus, invited mothers to participate were selected based on their accessibility. Prior to their participation, all mothers were required to give informed consent. It included the study’s objectives, the confidentiality of their information, the voluntary nature of their participation, and their right to withdraw at any time.

As there were two strategies for data collection, the mother gave informed consent through two procedures. In the first procedure, participants completed an online survey by themselves; in this method, mothers read the informed consent format, and if they wanted to participate, they were required to mark the option “Yes I accept”, and then they began to answer the survey. In the second procedure for data collection, mainly employed in rural areas, a printed version of the survey was administered by an intervention worker who later entered the answers into the online survey; in this data collection procedure, the informed consent was read by the worker, and if the participant accepted to participate, the worker marked the correspondent option in the printed version of the survey.

The study adhered to ethical standards outlined by the American Psychological Association and received approval from the university’s research ethics committee *[the committee’s statement code will be included in the final version after review]*.

### 2.2. Measures

#### 2.2.1. Infant Socioemotional Development Difficulties (BPSC)

The Spanish version of the Baby Pediatric Symptom Checklist (BPSC) [27] was administered. The BPSC is a brief instrument for the assessment of socioemotional difficulties in children under 18 months of age. The BPSC consists of 12 items distributed across three dimensions: “irritability”, “inflexibility”, and “difficulties with routines” [66]. The “irritability” dimension evaluates the caregiver’s perception of their child’s tendency to cry often, take a long time to calm down, or display frequent irritability. The “inflexibility” dimension includes items related to the child’s difficulties in adapting to changes, new people, new places, and their comfort level when held by individuals other than their primary caregiver. The “difficulties with routines” dimension assesses the caregiver’s perception of their child’s challenges in following routines, particularly those related to sleep [66]. Caregivers rate the frequency of their infant’s behaviors compared to most infants of the same age using a Likert scale ranging from 0 to 2 points. Previous studies have confirmed the effective function of this tool in Peruvian settings [67]. Confirmatory factor analysis (CFA) in the present study supported a three-factor structure (*χ*^2^(53) = 472.39, *p* < 0.001, CFI = 0.91, RMSEA = 0.09, SRMR = 0.05). Reliability analyses yielded Cronbach’s Alpha coefficients of 0.83 for “irritability”, 0.81 for “inflexibility”, and 0.81 for “difficulties with routines”.

#### 2.2.2. Mother’s Parental Stress (PSS)

The Parental Stress Scale (PSS) [68] was used to assess parents’ perceptions of the quantity and intensity of stress in their caregiver role. The scale consists of 12 items distributed across two dimensions: “baby reward” and “parental stress”. The “baby reward” dimension evaluates parents’ satisfaction in fulfilling their parental role, while the “parental stress” dimension addresses the stress parents experience. Participants rate these items on a Likert scale ranging from 1 to 5 points. Previous studies have confirmed the reliability and validity of the PSS in Peruvian settings [69]. Confirmatory factor analysis (CFA) conducted in the present study supported a two-factor structure (*χ*^2^(66) = 3054.54, *p* < 0.001, CFI = 0.90, RMSEA = 0.08, SRMR = 0.05). Reliability analysis indicated a Cronbach’s Alpha coefficient of 0.85 for the “parental stress” dimension and 0.82 for the “baby reward” dimension.

#### 2.2.3. Maternal Mental Health (GHQ-12)

The General Health Questionnaire [70] (GHQ-12) assesses the presence of psychopathological symptoms in both adolescents and adults. It comprises 12 items categorized into two dimensions: “general dysphoria” and “social dysfunction”. The first dimension encompasses items reflecting higher levels of anxiety and depression, while the second dimension encompasses items related to a decline in enjoyment and the ability to cope with everyday challenges. Respondents rate items on a Likert scale ranging from 0 to 3 points. Previous studies have confirmed the reliability and validity of the GHQ-12 in Peruvian populations [71]. For the purposes of this study, a CFA provided support for a two-factor solution (*χ*^2^(53) = 444.44, *p* < 0.001, CFI = 0.88, RMSEA = 0.09, SRMR = 0.05). The reliability was 0.79 for “general dysphoria” and 0.74 for “social dysfunction”.

### 2.3. Statistical Analyses

Statistical analyses using Structural Equation Modeling (SEM) were conducted in RStudio Statistical Software version 1.4.1106 (Boston, MA, USA) using the lavaan package version 0.6–8. The theoretical model was analyzed using the robust maximum likelihood method (MLR) estimator [72]. Model fit was assessed based on the following criteria: A comparative fit index (CFI) > 0.90, a root mean square error of approximation (RMSEA) < 0.08, and a standardized root mean square residual (SRMR) < 0.08.

The first tested model included the “baby reward” and “parental stress” as dimensions of PSS and a latent variable called Parental Stress. However, this initial model did not converge successfully. Consequently, only the “parental stress” dimension of the PSS was utilized as an observed variable in the final model.

To evaluate if the model was robust for the mother’s zone of residence (urban or rural) and the child’s sex, multigroup analyses were conducted. Three types of invariance were evaluated: configural invariance, in which the number of factors and the patterns of factor loadings were freely estimated for all groups; metric invariance, in which the factor loadings were constrained to be equal; scalar invariance, in which the intercepts were also constrained to be equal; and strict invariance, in which the unexplained variances of each item were constrained. To determine if these increasingly restrictive models differed significantly from each other, the difference in Chi-square was used as a criterion. Additionally, model equivalence was assessed using a CFI difference < 0.01, a RMSEA difference < 0.02, and a SRMR difference < 0.01.

## 3. Results

The study comprised 988 mothers aged between 18 and 42 years (*M* = 29.08, *SD* = 6.11). We note that 62.6% of the participants resided in rural settings, while 37.4% lived in urban areas. Most participants had completed secondary (57%) or primary (21.7%) education. Moreover, 79.5% reported being married or cohabiting with their partner. Nearly half of the participants (49.5%) were engaged in full-time family and childcare responsibilities, while 38.9% were employed.

The participants’ infants ranged in age from 6 to 18 months (*M* = 12.43, *SD* = 3.21), with 50.9% being male. Furthermore, 92.5% of the infants did not have developmental difficulties or disabilities, as was reported by mothers.

Regarding their socioeconomic status, 58.7% of the families were classified as belonging to a low socioeconomic level, and 41.3% were at a very low socioeconomic level. For a detailed overview of the sample’s sociodemographic characteristics, refer to Table 1.

Furthermore, Table 2 presents the descriptive data of the study variables and the bivariate correlations between them. The results indicate a strong association among children’s socioemotional development difficulties, including irritability, inflexibility, and difficulties with routines (0.54 < *r* < 0.68). Furthermore, general dysphoria (levels of anxiety and depression) shows moderate associations with the mother’s parental stress and the infant’s difficulties in following routines. Finally, the satisfaction reported by mothers in fulfilling their parental role (Baby Reward) demonstrates weak associations only with maternal depression and anxiety, as well as with children’s difficulties in following routines.

**Figure 2:**
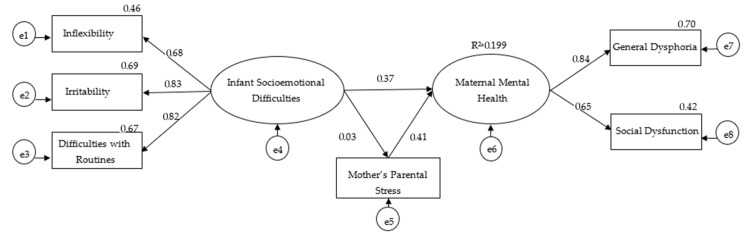
Structural equations model results.

About the results for the SEM analysis, all the fit indexes for the model were adequate: *χ*^2^(7) = 28.89, *p* < 0.001, CFI = 0.98, RMSEA = 0.06, and SRMR = 0.03. As shown in Figure 2, the results support the adequacy of the proposed model and establish that the infant socioemotional development difficulties were associated with maternal mental health directly and indirectly through the mother’s parental stress in this group of women living in socioeconomically vulnerable settings. Moreover, it is observed that 20% of the variability in maternal mental health difficulties is explained by infant socioemotional development difficulties and by the mother’s parental stress.

Table 3 and Table 4 present the results of the multigroup comparison for the sample according to the mother’s zone of residence and the sex of the infant. It is observed that, for both types of data, there are no problematic differences in the RMSEA, CFI, and SRMR indicators. Therefore, the invariance at the three levels for each is accepted.

## 4. Discussion

There is a clear relevance of perinatal mental health for the well-being of both women and the development of their children [11,12,13,14,15,16,17,18]. Additionally, existing evidence indicates a bidirectional relationship between maternal mental health and infant socioemotional development [73]. However, no studies have explored the effect of children’s characteristics and the parental role on maternal perinatal mental health in Peru. In light of the aforementioned considerations, this research focused on how infant socioemotional development difficulties are associated with maternal mental health through parental stress, complementing existing literature. This study was carried out in a sample of Peruvian mothers facing high socioeconomic vulnerability, a known risk factor for perinatal mental health issues [5].

The results obtained reveal that difficulties in infant socioemotional development are associated with maternal mental health problems during the perinatal period, both directly and through the mother’s parental stress. Consequently, the presence of child socioemotional difficulties would be associated with an increase in anxiety and depression, as well as a reduction in the capacity to enjoy and cope with daily challenges during the perinatal stage. Concurrently, the socioemotional difficulties of children place a significant burden on mothers, potentially leading to feelings of helplessness, emotional overload, and elevated parental stress, as was reported in previous literature [74]. As was reported, elevated parental stress, in turn, may impair the mothers, capacity to adequately cope with stressors related to motherhood [75], and this would result in unpleasant emotional states that would precede mental health problems [76]. It should be noted that these associations were found to be consistent for the urban and rural zones of the mother’s residence and for mothers of boys and girls.

These findings provide various crucial insights into maternal mental health challenges during the perinatal period in economically disadvantaged settings in Peru. Firstly, the greater prevalence of difficulties in infant socioemotional development in these groups [77,78] would create an increased demand for parenting tasks for these mothers due to the perception of high irritability, difficulty in following routines, and inflexibility that they perceive in their children. Secondly, responding to these demands may be more complicated for these mothers, given the scarcity of educational, economic, and support resources that they experience during the perinatal period. Finally, the greater biological, psychological, and social vulnerability of this period may increase parental stress [47]. Additionally, it can be posited that socioeconomic vulnerability may increase the risk of the emergence of mental health issues during the perinatal period, particularly in the context of the challenges associated with motherhood.

In the Peruvian context, parental stress and maternal mental health issues tend to become more pronounced due to social expectations of women as sole or primary caregivers [79]. These expectations could intensify pressures on mothers to meet caregiving demands, potentially worsening their perceived difficulties in their maternal role [76] and aggravating their mental health difficulties, particularly in economically disadvantaged backgrounds [80], where the birth and upbringing of young children exacerbate the gender imbalance [81,82].

Strengths of this study include its large and diverse sample of mothers from four regions and from rural and urban economically vulnerable sectors in Peru, which contains valuable knowledge on an understudied population. This provides clear evidence of the necessity for implementing policies and programs supporting parenting in these contexts, emphasizing early identification of infants’ socioemotional development difficulties and interventions that support the parental role of the mother while simultaneously attending to their emotional needs in order to prevent maternal stress and subsequent mental health problems. Thus, it is essential to develop interventions that address both the mother’s parental stress and their children’s socioemotional difficulties. In this context, there is substantial evidence indicating that interventions designed to specifically mitigate parental stress tend to have long-lasting, longitudinal effects in reducing psychopathological symptomatology in mothers [83].

Currently, in Peru, there are no interventions or policies addressing these challenges. This study provides evidence of the need for such public and/or private interventions that recognize the risks faced by mothers living in socioeconomically vulnerable settings during the transition to motherhood.

Additionally, another strength of the study is the nature and direction of the variables considered in the tested model. Firstly, the integration of variables related to the children and the maternal role in a single model to explain the emotional state of the mothers is noteworthy. Furthermore, it is important to have studied the relationships between child characteristics and parental roles in maternal mental health since previous literature has addressed the impact of maternal mental health on child development or has studied bidirectional relations between them.

Finally, another strength of this study relates to the use of a structural equation modeling (SEM) methodology, which allowed for the generation of robust evidence on the associations between the study variables by simultaneously considering the role of all the variables included in the model, as well as the measurement error.

Also, it is important to note that the present study has certain limitations that should be considered in future research. Firstly, the cross-sectional study design is a limitation because it does not allow for establishing predictive or causal relationships between the variables included in the model. Moreover, it is essential to acknowledge that all measures were self-reported by the mothers themselves, which may have introduced biases in the responses. Therefore, it would be beneficial for future studies to include the participation of other reporters, such as fathers or other caregivers, who can provide information about the children and/or their mothers. Another significant improvement to the measurement employed would be the use of direct observational measures of the children’s socioemotional development or of the mother’s stress levels. This would enable an “objective” measure of the children’s socioemotional development and/or the mother’s health.

Similarly, the tested model does not incorporate other variables that could contribute to an explanation of maternal perinatal health reported in previous literature. These include potential confounding variables such as parity, mode of delivery, comorbidities, the role of other caregivers (if any), the father’s involvement in the care of the children [84], or the quality of care provided to children. Furthermore, the extent to which fathers assume co-parenting responsibilities [85] and the emotional support they can provide to mothers during the perinatal period [86] need to be assessed in future research as well.

Additionally, the study’s exclusive focus on a socioeconomically vulnerable group limits the ability to generalize these findings to populations living in higher socioeconomic conditions. It remains uncertain whether similar relationships between infant socioemotional development, parental stress, and maternal mental health exist in more advantaged groups or if these associations may be less pronounced or absent altogether. While studying a socioeconomically disadvantaged group has provided valuable insights not previously explored in the literature, the absence of studies involving medium socioeconomic levels in Peru prevents meaningful comparisons of these results across broader socioeconomic contexts. Future research should aim to include diverse socioeconomic strata to better understand how these dynamics vary across different population segments and to ensure comprehensive insights into maternal mental health across Peru.

Finally, it is important to acknowledge the potential for bias within the study group, which consisted of mothers of infants receiving an intervention to improve infant development in socioeconomically vulnerable children. The challenges concerning the mother’s condition are in determining the true nature of relationships between variables in socioeconomically vulnerable groups who lack access to such interventions.

## 5. Conclusions

The present study aimed to test a model that associated perinatal maternal mental health with infant socioemotional difficulties and a mother’s parental stress in a socioeconomically vulnerable sample of Peru. It was found that perinatal maternal mental health was directly associated with infant socioemotional development difficulties and a mother’s parental stress. It was also found that maternal parental stress mediated the relationship between infant socioemotional difficulties and perinatal mental health. The findings underscore the necessity for interventions aimed at the early detection of socioemotional difficulties in children and high levels of parental stress, with the objective of preventing their adverse effects on the perinatal mental health of mothers living in conditions of socioeconomic vulnerability. This research is novel in Peru and Latin America because the model associates maternal mental health with parental and child variables, and the study was conducted in a group with high socioeconomic vulnerability from urban and rural areas in four Peruvian regions.

## Figures and Tables

**Table 1:** Mothers and infants’ sociodemographic information.

Variable	
Mothers	
Age in years: *M*/*SD*	29.08/6.11
Living area: *n*/*f*	
Urban	370/37.4
Rural	618/62.6
Educational level: *n*/*f*	
No schooling	70/7.1
Completed elementary school	214/21.7
Completed secondary school	563/57
Completed non-university degree	81/8.2
Graduate degree	59/6
Postgraduate	1/0.1
Civil status: *n*/*f*	
Married or cohabiting	785/79.5
Single	92/9.3
Divorced, widowed, or separated	111/11.2
Occupation: *n*/*f*	
Employed	384/38.9
Student	46/4.7
Full-time care of family and children	489/49.5
Unemployed	39/3.9
Others	30/3
Infants	
Age in months: *M*/*SD*	12.43/3.21
Gender: *n*/*f*	
Girls	485/49.1
Boys	503/50.9
Disability: *n*/*f*	
Yes	74/7.5
No	914/92.5
Socioeconomic Status: *n*/*f*	
Low	580/58.7
Very low	408/41.3

**Table 2:** Descriptive statistics and bivariate correlation coefficients between main variables.

	1	2	3	4	5	6	*M*	*SD*	Range
1. Inflexibility							5.7	1.9	4–12
2. Irritability	0.58 ***						6.3	2	4–12
3. Difficulties with Routines	0.54 ***	0.68 ***					5.6	1.8	4–12
4. Mother’s Parental Stress	0.08 *	0.15 ***	0.17 ***				2.4	0.9	1–5
5. Baby Reward	−0.00	−0.05	−0.07 *	−0.01			4.4	0.8	1–5
6. General Dysphoria	0.21 ***	0.22 ***	0.32 ***	0.34 ***	−0.09 **		8.8	3	6–21
7. Social Dysfunction	0.12 ***	0.15 ***	0.20 ***	0.30 ***	−0.06	0.54 ***	10	2.9	6–23

Note. * *p* < 0.05, ** *p* < 0.01, *** *p* < 0.001.

**Table 3:** Test of invariance for the model by urban and rural zone of residence.

Model	*X* ^2^	*gl*	RMSEA	CFI	SRMR	ΔRMSEA	ΔCFI	ΔSRMR
Urban Model	1317.41	15	0.08	0.99	0.03			
Rural Model	1987.61	15	0.03	0.99	0.01			
Configural Invariance	3314.33	30	0.05	0.99	0.02			
Metric Invariance	3314.35	30	0.05	0.99	0.02	0.00	0.01	0.00
Scalar Invariance	3314.35	30	0.05	0.99	0.03	0.00	0.00	0.01
Latent Mean Invariance	3314.35	30	0.05	0.99	0.03	0.01	0.00	0.00

**Table 4:** Test of invariance for the model by infant’s sex.

Model	*X* ^2^	*gl*	RMSEA	CFI	SRMR	ΔRMSEA	ΔCFI	ΔSRMR
Male Model	1741.12	15	0.08	0.99	0.02			
Female Model	1549.02	15	0.07	0.99	0.02			
Configural Invariance	3290.82	30	0.05	0.99	0.02			
Metric Invariance	3290.82	30	0.06	0.99	0.02	0.00	0.00	0.01
Scalar Invariance	3290.82	30	0.05	0.99	0.03	0.00	0.00	0.01
Latent Mean Invariance	3290.82	30	0.05	0.99	0.03	0.00	0.00	0.00

## Data Availability

The study data for replicating this research and their analyses are not publicly available but can be accessed by reasonable request to the corresponding author Correspondence regarding this article should be addressed to Magaly Nóblega, Pontifical Catholic University of Peru, 1801 Universitaria Avenue, 15088, mnoblega@pucp.pe.
